# DNA Unwinding by Ring-Shaped T4 Helicase gp41 Is Hindered by Tension on the Occluded Strand

**DOI:** 10.1371/journal.pone.0079237

**Published:** 2013-11-08

**Authors:** Noah Ribeck, Omar A. Saleh

**Affiliations:** 1 Department of Physics, University of California Santa Barbara, Santa Barbara, California, United States of America; 2 Department of Materials and Biomolecular Science and Engineering Program, University of California Santa Barbara, Santa Barbara, California, United States of America; German Cancer Research Center, Germany

## Abstract

The replicative helicase for bacteriophage T4 is gp41, which is a ring-shaped hexameric motor protein that achieves unwinding of dsDNA by translocating along one strand of ssDNA while forcing the opposite strand to the outside of the ring. While much study has been dedicated to the mechanism of binding and translocation along the ssDNA strand encircled by ring-shaped helicases, relatively little is known about the nature of the interaction with the opposite, ‘occluded’ strand. Here, we investigate the interplay between the bacteriophage T4 helicase gp41 and the ss/dsDNA fork by measuring, at the single-molecule level, DNA unwinding events on stretched DNA tethers in multiple geometries. We find that gp41 activity is significantly dependent on the geometry and tension of the occluded strand, suggesting an interaction between gp41 and the occluded strand that stimulates the helicase. However, the geometry dependence of gp41 activity is the opposite of that found previously for the *E. coli* hexameric helicase DnaB. Namely, tension applied between the occluded strand and dsDNA stem inhibits unwinding activity by gp41, while tension pulling apart the two ssDNA tails does not hinder its activity. This implies a distinct variation in helicase-occluded strand interactions among superfamily IV helicases, and we propose a speculative model for this interaction that is consistent with both the data presented here on gp41 and the data that had been previously reported for DnaB.

## Introduction

Ring-shaped, hexameric helicases are motor proteins responsible for unwinding parental DNA during replication. The ring-shaped hexamer is a widely conserved motif of replicative helicases across all forms of life [1]. Typically, replicative helicases encircle and translocate along one ssDNA strand while forcing the other strand to the outside of the ring, a process termed ‘steric occlusion’ [2]–[4]. As the component at the front of the replication machinery, it is essential for the speed of the replicative helicase to be finely controlled such that DNA unwinding proceeds in coordination with the synthesis of both leading and lagging strands. In fact, the speed of one particular ring-shaped helicase has been shown to be highly tunable: DnaB from *E. coli* is capable of a large range of unwinding rates depending on the presence of other components of the replication machinery [5]. This suggests that its speed is regulated by interaction with other parts of the replisome.

The precise mechanical or biochemical mechanism that controls helicase unwinding rate is not well understood. In order to elucidate the nature of this regulation, we must fully understand the details of how the helicase interacts with other replication proteins and with single- and double-stranded DNA (ssDNA and dsDNA).

Indeed, recent work has suggested that altered DNA geometry can modulate the speed of *E. coli* DnaB. By mechanically manipulating single DNA molecules in different orientations, it was shown that DnaB's unwinding rate is slowed when the ssDNA tails are pulled directly apart, compared to when only one strand is constrained and the other is free [6]. This suggested that the unwinding rate could be tuned by controlling the proximity of the occluded strand to the outside of the hexameric ring. This finding raises the question of whether a DNA geometry-dependent unwinding rate is a common feature of replicative helicases.

In this work, we perform similar single-molecule measurements with another superfamily IV hexameric helicase, gp41 from bacteriophage T4. We find that at low force, the DNA geometry-specific effect seen in DnaB is not present in gp41. Furthermore, we describe the surprising result that high tension on the occluded strand hinders unwinding by gp41, which did not occur with DnaB, suggesting that despite their similarities, these hexameric helicases interact with the replication fork in different ways that have significant effects on their speeds.

## Materials and Methods

Helicase gp41 was expressed and purified as described in [7]. The hairpin with a 389 bp dsDNA stem and the 3′ fork with a 5322 bp dsDNA region were constructed and tethered in glass flow cells as described in [6]. Helicase was added to the flow cell at 100 nM monomer concentration in gp41 buffer [8], [9]: 25 mM Tris-acetate (pH 7.5), 150 mM potassium acetate, 10 mM magnesium acetate, 1 mM DTT, and 5 mM ATP. Data were collected in real time at 60 Hz using multiplexed magnetic tweezers [10], [11]. Forces were determined and rates of helicase activity were extracted from the data as described in [6]. All experiments were conducted at 22°C.

## Results

We use two different single-molecule DNA tethering schemes to measure the activity of T4 gp41 helicase. By using multiple DNA geometries, we can effectively modulate the orientation of the replication fork DNA with respect to the helicase, as was done with *E. coli* DnaB [6]. We compare the helicase unwinding rate in each assay to determine the effect of DNA geometry on helicase motion. In particular, we use magnetic tweezers to manipulate paramagnetic beads tethered by different DNA substrates, and use the measured bead position to report on helicase unwinding.

**Figure 1:**
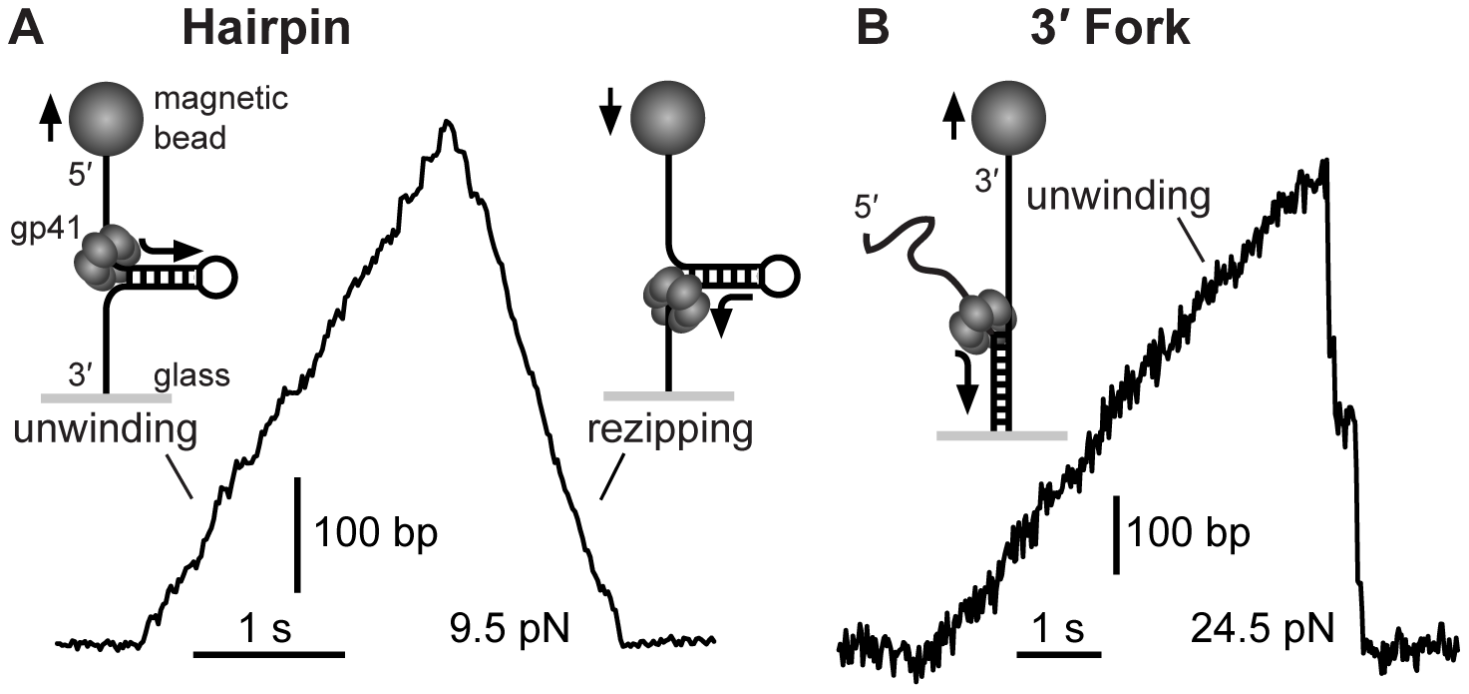
Example trajectories of single-molecule helicase events by gp41. (A) In the hairpin geometry, gp41 first unwinds the dsDNA hairpin stem (389 bp), then proceeds to translocate along ssDNA while the hairpin stem reanneals in its wake. (B) In the 3′ fork geometry, gp41 unwinds the tethered dsDNA (5322 kb). When the helicase unbinds from the substrate, the dsDNA reanneals to its original state.

The first geometry we use is a tethered hairpin (Figure 1A). The tethered hairpin assay provides a way to measure the speed of a single hexameric helicase both while unwinding dsDNA and while translocating along ssDNA [6], [8], [12], [13]. In this assay, a DNA hairpin is tethered between a glass surface and a magnetic bead through two ssDNA tails. When a gp41 hexamer binds to the 5′ tail, it unwinds the hairpin stem, and then proceeds to translocate along ssDNA while the hairpin reanneals in its wake. By tracking the position of the magnetic bead while the hairpin opens and closes, we can measure the position of the helicase along the hairpin as a function of time, which provides a measurement of both the dsDNA unwinding rate and ssDNA translocation rate.

**Figure 2:**
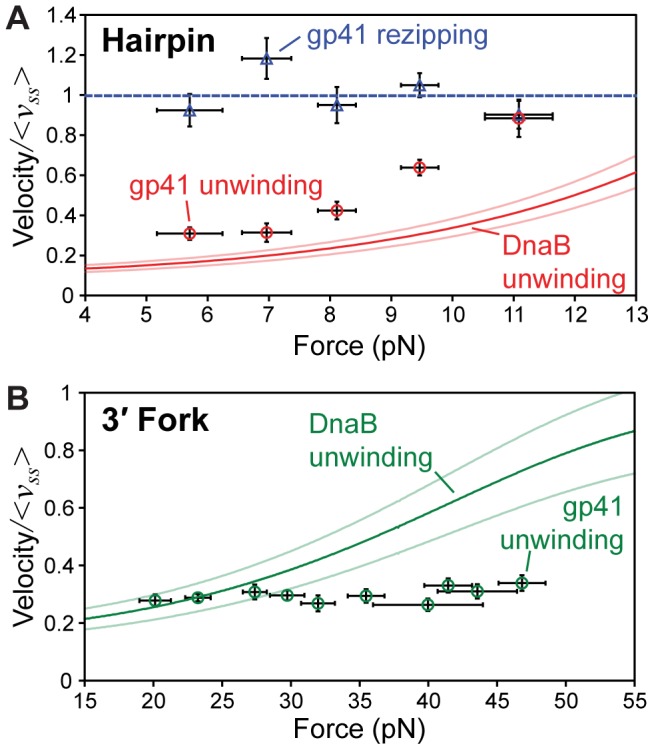
Velocities of gp41 in each tethered DNA geometry. (A) Hairpin unwinding and rezipping rates (mean ± SE) measured at various forces (± SD). The force-independent mean rezipping rate (<*v_ss_*> = 409±16 bp/s) is equivalent to the translocation rate of gp41 on ssDNA. (B) Unwinding rates (mean ± SE) measured at various forces (± SD) in the 3′ fork assay. In both panels, velocities are plotted as a fraction of <*v_ss_*> as measured in the hairpin assay. For comparison, best-fit curves and approximate standard error of the active/passive helicase model [14], [15] to identical measurements of DnaB unwinding from [6] are shown (SE ∼13% for hairpin and ∼17% for 3′ fork). Relative to its <*v_ss_*>, gp41 unwinding is faster than DnaB in the hairpin assay, while DnaB unwinding is faster than gp41 in the 3′ fork assay. In both cases, these differences are greater at higher forces.

We observed a total of 28 events of complete unwinding and reannealing with gp41 in the hairpin assay at various forces. The rates of helicase activity computed from these events are shown in Figure 2A. Pauses in helicase activity were occasionally observed, but were removed from the analysis before computing the mean rates reported here. As expected for a ‘passive’ helicase [14]–[17], the unwinding rate increases with applied force due to decreasing stability of the dsDNA, which increases basepair opening fluctuations that the helicase exploits to move forward. During hairpin rezipping, the rate of gp41 activity is independent of the applied force, which is consistent with the interpretation that the hexamer is simply translocating along ssDNA during this phase, and is therefore not sensitive to basepair stability [6], [8]. These data are quantitatively identical to previous single-molecule measurements of gp41 by Lionnet *et al* [8].

The 3′ fork assay (Figure 1B) is an alternative way to measure the helicase unwinding rate [6], [18]. In this assay, dsDNA is tethered at one extremity to a glass surface, while the other end is forked with two ssDNA tails, of which the 3′ end is tethered to a magnetic bead, and the 5′ end is free; thus, tension is applied between the dsDNA and the 3′ tail. When a gp41 hexamer binds to the free 5′ tail, it proceeds to unwind the dsDNA. When high forces are applied (>20 pN), the end-to-end extension of ssDNA is significantly greater than that of dsDNA, and thus helicase unwinding is coupled to upward bead motion. By tracking the position of the bead and removing pauses, we can then measure the unwinding rate. Attempts to measure gp41 activity with the 5′ tail tethered to the bead did not yield any unwinding events.

We observed a total of 104 unwinding events with gp41 in the 3′ fork assay at various forces. The pause-removed unwinding rates computed from these events are shown in Figure 2B. Unlike on the hairpin, we observe very little variation in the unwinding rate in this geometry, despite the wide range of forces used. Like the hairpin assay, increasing force destabilizes the basepairs in the 3′ fork geometry, but this destabilization results in no measurable increase in the speed of gp41 unwinding. This result is in contrast to measurements of *E. coli* DnaB, whose unwinding rate markedly increased with force in both DNA geometries [6].

In the prevailing model of passive helicase activity, the unwinding rate depends partially on rapid fluctuations of basepairs between the opened and closed states [14]–[17]. Therefore, to directly compare gp41 unwinding rates between the two assays, we must account for the effect of the applied force on the equilibrium basepair stability in each assay. To destabilize dsDNA by an equivalent amount, it takes a greater applied force in the 3′ fork geometry (where the force shears the basepairs) than it does in the hairpin geometry (where the force pulls the basepairs directly apart). For each assay, the basepairing free energy can be expressed as a function of force by integrating the force-extension curves [19], as was done in [6], [8], [20]. For the 3′ fork geometry, basepairing free energy is calculated for duplexes attached on each end by a single strand, where dsDNA melts at ∼65 pN [21].

**Figure 3:**
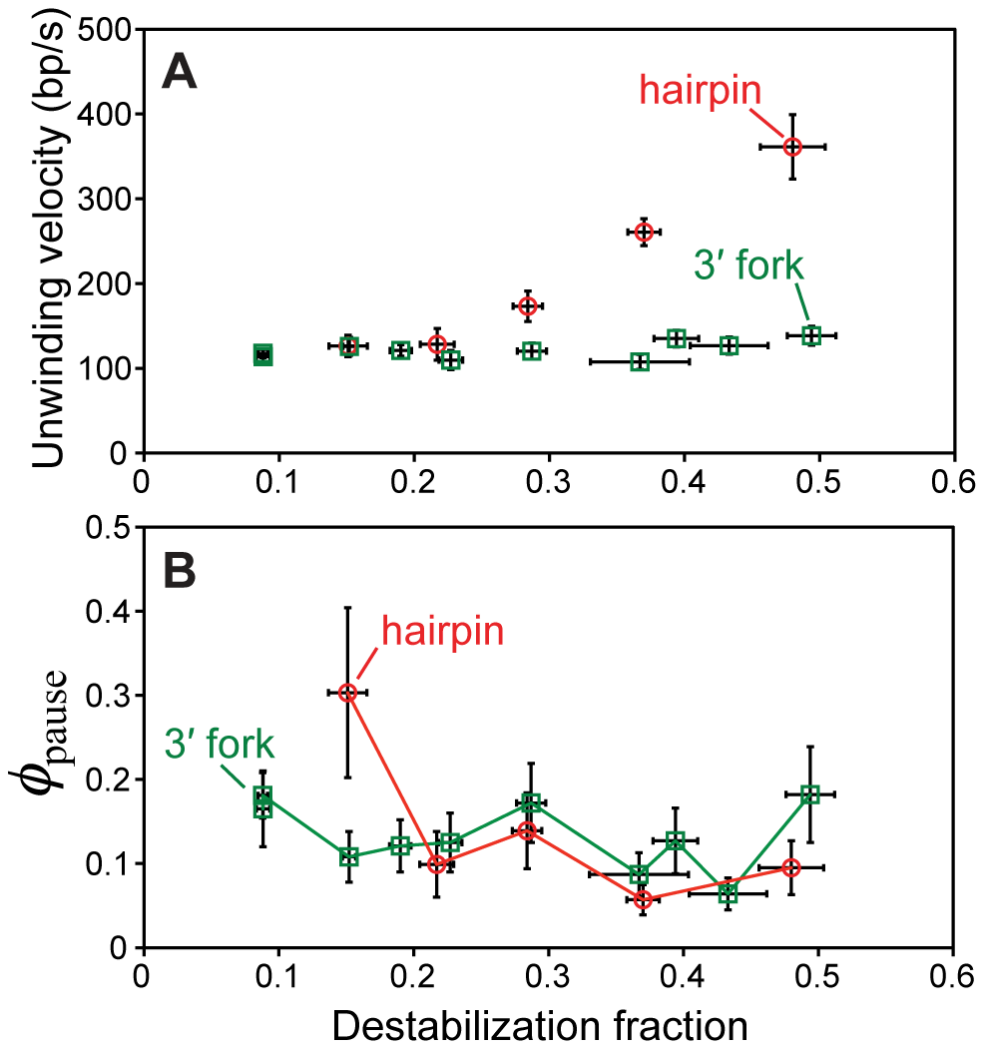
Unwinding and pausing activities of gp41 in each tethered DNA geometry. Data are plotted as a function of destabilization fraction (the degree to which dsDNA is destabilized by force, ranging from 0 to 1; ± SD). (A) Unwinding rates of gp41 measured in each assay (same data shown in Figure 2; ± SE). (B) ***φ***_pause_, the fraction of time spent pausing by gp41 while bound to the DNA substrate (mean ± SE).

To allow for direct comparison of different tethered DNA geometries, we consider the gp41 unwinding rates at comparable values of the equilibrium basepair stability, since this is the parameter that directly affects helicase velocity [14], [15]. In Figure 3A, the unwinding rate data on the hairpin and 3′ fork are re-plotted as a function of the extent to which the basepairs are destabilized. At low destabilization (corresponding to the lower forces in each assay), the unwinding rates in each assay are identical, indicating that in this regime, the unwinding rate of gp41 is not dependent on the tethered DNA geometry. However, when the basepairs are more highly destabilized, unwinding of the hairpin is much faster than unwinding of the 3′ fork, which stays flat over the entire measured force range. This behavior is different from that observed in DnaB, which is consistently faster on the 3′ fork at all values of the basepair stability [6].

As previously mentioned, processive gp41 activity was occasionally interrupted by periods of pausing without unbinding followed by continued activity. This is a common feature of motor protein activity [22], [23], and has been observed previously in hexameric helicases [6], [20]. The prominence of this pausing activity can be quantified by computing the amount of time spent in this pause state, as a fraction of the total time spent bound to the DNA substrate. In this work, gp41 was in a pause state approximately 10–15% of the bound time, a value that is consistent in both DNA geometries over the entire force ranges (Figure 3B). This is different from DnaB, whose pausing activity is dependent on both DNA geometry and force [6]. This provides further evidence that the means of interaction with the replication fork DNA differs between DnaB and gp41.

## Discussion

Single-molecule assays are excellent means for measuring the activity of motor proteins as they travel along their substrates, as they permit direct quantification of motor trajectories, and enable the use of force as an independent experimental parameter. However, in the case of helicases and forked DNA substrates, this approach is complicated by the geometry of the tethered DNA: tension can be applied across any two of the fork's three termini (5′ tail, 3′ tail, and dsDNA), and this choice can affect both the stability of basepairs against the force, and the helicase/DNA interaction.

In previous work, we showed that the activity of the bacterial helicase DnaB is dependent on the DNA geometry [6], while the measurements reported here show that the T4 phage helicase gp41 also has a DNA geometry-dependent activity. In both cases, the geometry-dependent activity remains even after accounting for the effect of force on basepair stability. However, while the assays utilized were identical, the nature of the geometry-dependent activity differs between DnaB and gp41. These observations offer two conclusions about the biophysics of replicative helicases. First, the interactions between the helicase and the forked DNA substrate play a role in determining the rate of unwinding and frequency of pausing. Second, the mechanism of this interaction is not conserved over all replicative helicases, despite their structural similarities.

The measurements of gp41 reported here are directly compared to the unwinding activity of DnaB from [6] in Figure 2. We observe that gp41 unwinding is faster (relative to its translocation rate on ssDNA) than DnaB at all forces in the hairpin assay, while the inverse is true for the 3′ fork assay. In both cases, the difference in relative velocities between the two motors increases at higher forces. In fact, gp41 unwinding in the 3′ fork assay is so much slower than DnaB at high forces, the expected increased unwinding rate with increasing assisting force is completely absent. This indicates that the geometry of forces in the 3′ fork assay result in significantly suboptimal gp41 unwinding. Inversely, the geometry of forces in the hairpin assay results in suboptimal DnaB unwinding.

The molecular mechanism by which force hinders helicase activity must be interpreted using the differences between the particular geometries used. One difference between the hairpin and 3′ fork assay is that the encircled strand is under tension only in the hairpin geometry. However, because the translocation rate (as measured by hairpin rezipping) is force-independent for both gp41 (Figure 1A) and DnaB [6], we conclude that the tension on the encircled strand in the hairpin assay does not affect helicase translocation. Indeed, our work on DnaB showed that tension on the encircled strand only affects helicase activity at very high forces (above 20 pN), likely due to the compacted nature of the encircled ssDNA [6]; this compaction has since been confirmed by crystal structures [24].

Instead, a likely mechanism for the variation of helicase activity between the assays lies in the differing force on the occluded 3′ strand. In particular, we posit that both helicases have stimulating interactions with the occluded strand, and that these biomolecular interactions can be disrupted by force applied to that strand, slowing the helicase. Indeed, there is evidence that DnaB unwinding is stimulated by an occluded strand interaction [25], and our prior work concluded that this interaction is force-sensitive [6]. It has also been suggested that the occluded strand binds to the outside of two or three subunits of the gp41 hexamer [26], [27].

While we posit that both DnaB and gp41 are stimulated through force-sensitive occluded-strand interactions, those interactions cannot be identical: this is indicated by the reversal in the relative rates of the two helicases between the two assays. This reversal leads us to discard arguments that posit only a different strength of occluded-strand interaction between the two helicases: if binding affinity were the only difference, and since force is expected to decrease affinity, we would expect both helicases to go slower in the 3′ fork geometry, where the higher force would more strongly destabilize the occluded-strand interactions. This disagrees with the data, particularly the increased unwinding rate of DnaB in the 3′ fork geometry. Thus, while affinity could play a role, we argue it must be secondary.

Instead, we speculate the reversal in rates could be due to differences between the two helicases in the relative *orientation* of the helicase/occluded-strand complex: such differences would impart on the complex a different stability against different orientations of applied force. It is well established that the biomolecular effect of tension is best understood as a vectorial, and not a scalar, quantity. For example, dsDNA itself can be destabilized by relatively small forces (∼15 pN) when a pair of proximal 3′ and 5′ ends are pulled directly apart in a peeling geometry, while much larger destabilization forces (∼65 pN) are required when a distal pair are pulled apart across a long dsDNA molecule in a shearing geometry [19]. We must therefore consider both orientation and magnitude of the applied force in any model of helicase/occluded strand interactions.

We thus speculate that, while both gp41 and DnaB are stimulated by an occluded strand interaction, their differing force-dependent activities are likely due to differing orientations of the occluded strand relative to the hexamer. In particular, we posit that, for DnaB, the occluded strand is bound to the outside of the ring in an orientation that is roughly parallel to the ring's central channel. For gp41, we posit the occluded strand is bound to the outside of the ring in an orientation that is roughly perpendicular to the ring's central channel.

These interactions would be tested differently by the hairpin assay (where the force is more perpendicular to the central channel) and the 3′ fork assay (where the force is more parallel to the central channel). For DnaB, the 3′ fork assay would lead to a shear force on the occluded strand interaction, since the force and the occluded strand would be roughly parallel. As discussed, shear geometries are better withstood by biomolecular bonds; thus, we expect that the interaction will persist in the 3′ fork geometry, stimulating DnaB activity. In contrast, in the hairpin assay the force would be perpendicular to the occluded strand, leading to a peeling geometry. As peeling geometries are mechanically weak, we expect that the interaction will be easily disrupted, explaining the relatively slow unwinding of DnaB in the hairpin geometry.

This model explains the reversal of relative activity for gp41 compared to DnaB. In the 3′ fork assay, the force is parallel to the central channel, which is perpendicular to the gp41-bound occluded strand. This will cause the gp41/occluded strand interaction to be peeled off relatively easily, hindering unwinding activity, as observed. In the hairpin assay, the force and occluded strand are more parallel, leading to a shear geometry against which the occluded strand interaction is more stable. Thus, the interaction is expected to persist, resulting in faster unwinding by gp41.

How could gp41 bind the occluded strand to result in a perpendicular orientation relative to the central ring? One possibility is found in the Steric Exclusion and Wrapping (SEW) model, recently proposed by Graham *et al*. for the eukaryotic hexameric helicase MCM [28]. In this picture, the occluded strand wraps around and stabilizes the hexameric ring; the resulting orientation is indeed perpendicular, as shown in [28], [29]. A wrapping geometry has also been shown for the hexameric *E. coli* transcription termination factor Rho, which wraps RNA around its exterior [30], [31]. On the other hand, for DnaB, there is evidence against ssDNA wrapping around the hexamer [32], consistent with our interpretation here.

We present this model as a hypothesis based on the conclusion that the geometry-dependent activity of gp41 is the inverse of that of DnaB between the two identical assays. In this picture, both helicases interact with the occluded strand in a way that stimulates their unwinding activities. For DnaB, we posit the occluded strand simply lies along the ring, parallel to the central channel. For gp41, we posit that this interaction involves partial wrapping of the occluded strand around the hexamer, giving a perpendicular orientation to the occluded strand relative to the central channel. We note that an interaction of the occluded strand with more than one monomer of gp41, as posited by other authors [26], [27], implies a partially-wrapped, roughly perpendicular orientation of the occluded strand, assuming a ring-shaped hexamer. In this model, and in the hairpin assay, torque generated on gp41 between forces at the ring exterior and central channel will tend to rotate the complex and align those points; while this will alter the orientation of the applied force, we suggest enough shear orientation will be retained to strengthen the interaction in this geometry.

This picture results in opposite behavior for each of the helicases in the two tethered DNA geometries. In the hairpin assay, the force on the occluded strand is perpendicular to the central channel, which disrupts its complex with DnaB. On the other hand, in the 3′ fork assay, the force on the occluded strand is parallel to the central channel, which disrupts its complex with gp41.

## Conclusions

The measurements reported here show that hexameric helicase unwinding activity on a tethered DNA substrate is highly dependent on the geometry of applied force. Further, we have established that the geometry-dependent nature of T4 gp41 helicase activity is different from that of previously studied *E. coli* DnaB [6]. These results underscore the complexity of even nominally simple single-molecule measurements, at least in the application to hexameric helicases; previous studies have not considered the kinds of geometry-dependent effects we have observed [8], [20].

Here, we have interpreted the present and previous [6] data on the variations in gp41 and DnaB unwinding rate with DNA geometry using a speculative model that focuses on the helicase/occluded-strand interaction. Apart from explaining our single-molecule data, this model is directly supported by known facts of the DnaB-occluded strand interaction [32], the gp41 occluded strand interaction [26], [27], and more indirectly supported by analogies to DNA-wrapping interactions by other hexameric helicases [28]–[31]. In this picture, the two hexamers interact with the ssDNA tail that is occluded to the outside of the ring in a way that stimulates their unwinding activity. However, we suggest that gp41 and DnaB have different orientations with respect to the occluded strand, which leads to a variation in the disruption of the occluded-strand interaction with orientation of the applied force, and thus different unwinding activities on tethered DNA structures of different geometries. We posit that these differences are due to partial wrapping of the occluded strand by gp41, but not by DnaB.

The ssDNA tails in these single-molecule assays represent the unsynthesized leading and lagging strands of the replication fork. The notion that unwinding and pausing of the helicase can be altered by various means of interaction with the occluded strand (the leading strand — for 5′ to 3′ motors like gp41 and DnaB) raises the possibility that modulation of the orientation and tension of the occluded strand could regulate the speed of the helicase, perhaps to maintain replisome coordination. However, the present results on gp41 make it clear that any DNA-mediated regulatory mechanism of helicase activity is unlikely to be universal. The most striking difference between the replication machinery in the two systems discussed here is that the *E. coli* replisome contains a protein that physically connects the helicase to both the leading and lagging polymerases (the tau complex) [33], while the T4 replisome does not. Perhaps, then, it is sensible that T4 would require a more prominent occluded strand interaction for its activity to be effectively controlled by those means. Further study of the geometry-dependent unwinding activity of complete replisomes will elucidate the difference between mechanistic interactions of different replication systems. Here, we have reported a first step in that direction, by probing the helicase-DNA interaction in isolation.

## Acknowledgments

The authors gratefully acknowledge Michelle Spiering and Stephen Benkovic for helpful discussions and for donating protein.
